# Study of the in vivo role of Mce2R, the transcriptional regulator of *mce2* operon in *Mycobacterium tuberculosis*

**DOI:** 10.1186/1471-2180-13-200

**Published:** 2013-09-05

**Authors:** Marina Andrea Forrellad, María Verónica Bianco, Federico Carlos Blanco, Javier Nuñez, Laura Inés Klepp, Cristina Lourdes Vazquez, María de la Paz Santangelo, Rosana Valeria Rocha, Marcelo Soria, Paul Golby, Maximiliano Gabriel Gutierrez, Fabiana Bigi

**Affiliations:** 1 Instituto de Biotecnología, CICVyA-INTA, N. Repetto and De los Reseros, Hurlingham 1686, Argentina; 2 Animal Health Veterinary Laboratories Agency (Weybridge), New Haw, Addlestone, Surrey KT15 3NB, UK; 3 Research Group Phagosome Biology, Helmholtz Centre for Infection Research, Inhoffenstrasse 7, Braunschweig 38124, Germany; 4 Microbiología Agrícola Facultad de Agronomía, Universidad de Buenos Aires, Avenue San Martín 4453, Buenos Aires 1417, Argentina; 5 Present address: Division of Mycobacterial Research, MRC National Institute for Medical Research, The Ridgeway, Mill Hill, London NW7 1AA, UK

**Keywords:** *Mycobacterium tuberculosis*, *mce2R*, Phagosome arresting

## Abstract

**Background:**

Tuberculosis is one of the leading causes of mortality throughout the world. *Mycobacterium tuberculosis*, the agent of human tuberculosis, has developed strategies involving proteins and other compounds called virulence factors to subvert human host defences and damage and invade the human host. Among these virulence-related proteins are the Mce proteins, which are encoded in the *mce1*, *mce2*, *mce3* and *mce4* operons of *M. tuberculosis*. The expression of the *mce2* operon is negatively regulated by the Mce2R transcriptional repressor. Here we evaluated the role of Mce2R during the infection of *M. tuberculosis* in mice and macrophages and defined the genes whose expression is *in vitro* regulated by this transcriptional repressor.

**Results:**

We used a specialized transduction method for generating a *mce2R* mutant of *M. tuberculosis* H37Rv. Although we found equivalent replication of the MtΔmce2R mutant and the wild type strains in mouse lungs, overexpression of Mce2R in the complemented strain (MtΔmce2RComp) significantly impaired its replication. During *in vitro* infection of macrophages, we observed a significantly increased association of the late endosomal marker LAMP-2 to MtΔmce2RComp-containing phagosomes as compared to MtΔmce2R and the wild type strains. Whole transcriptional analysis showed that Mce2R regulates mainly the expression of the *mce2* operon, in the *in vitro* conditions studied.

**Conclusions:**

The findings of the current study indicate that Mce2R weakly represses the *in vivo* expression of the *mce2* operon in the studied conditions and argue for a role of the proteins encoded in Mce2R regulon in the arrest of phagosome maturation induced by *M. tuberculosis*.

## Background

Tuberculosis is still one of the leading causes of mortality throughout the world. The HIV/AIDS pandemic, the deterioration of public health systems in developing countries, and the emergence of multi-drug resistance of untreatable forms of tuberculosis have further contributed to that spread. Infection by the causative agent of tuberculosis, *Mycobacterium tuberculosis*, is achieved by strategies involving uptake and replication of the bacterium in host macrophages and the weakening or modification of the host immune response [1,2]. In recent years, there has been a considerable advance in the understanding of the molecular bases of pathogenicity, virulence and persistence of mycobacteria. One significant contribution to this knowledge has been the identification of essential proteins for mycobacterial virulence. The Mce (*mammalian cell entry*) proteins are a group of secreted or surface-exposed proteins encoded by *mce* genes. These genes are situated in operons, comprising eight genes, organized in exactly the same manner. *M. tuberculosis* has four *mce loci*: *mce1*, *mce2*, *mce3* and *mce4*. The name of these proteins is derived from the function firstly assigned to Mce1, related to the ability of mycobacteria to enter mammalian cells and survive inside macrophages [3]. *mce* operons with an identical structure have been identified in all *Mycobacterium* species examined, as well as in other species of Actinomycetales [4]. A considerable number of studies have demonstrated that Mce proteins are related to the virulence of each member of the *M. tuberculosis* complex. Flesselles et al. [5] have reported that a BCG strain mutated in *mce1* exhibits a reduced ability to invade the non-phagocytic epithelial cell line HeLa. Sassetti and Rubin [6] have then found that *mce1* disruption causes attenuation of *M. tuberculosis*. Further studies have shown that a strain knockout in *mce1* has reduced ability to multiply when inoculated by the intratracheal route in mice. However, the same *mce1* mutant strain is hypervirulent when inoculated intraperitoneally in mice. Moreover, Shimono et al. [7] have demonstrated that a strain of *M. tuberculosis* mutant in the *mce1* operon can kill mice more rapidly than the wild type strain after intravenous inoculation. Variations in the level of virulence depending on the route of bacterial inoculation have also been observed in mutants of the *mce2* and *mce3* operons when assessed in mice [8,9], suggesting that *M. tuberculosis* regulates the expression of Mce proteins to adapt to the variety of environmental host conditions. Consistently with this presumption, regulatory proteins that control the transcription of *mce1*, *mce2* and *mce3* have been identified in *M. tuberculosis*.

In a previous study, we have demonstrated that *mce2R* (*Rv0586*), the first open reading frame of the *mce2* operon, encodes for a *mce2*-specific GntR transcriptional repressor [10]. This regulator poorly controls the expression of Mce2 proteins during the *in vitro* growth of *M. tuberculosis* in rich media [10], suggesting that Mce2R control the expression of *mce2* when the bacteria encounter a particular growth-restricted environment. In order to test this possibility, in this study we compared the replication of *M. tuberculosis* in mice in the absence and in the presence of Mce2R. The genes regulated by Mce2R and the role of this regulator in the maturation of the *M. tuberculosis*-containing phagosomes in macrophages was also investigated.

## Results

### Deletion of *mce2R* in *M. tuberculosis*

**Figure 1:**
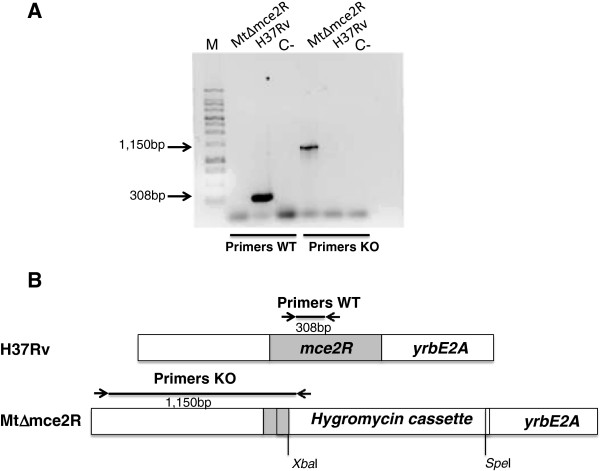
**Deletion of *mce2R *from *M. tuberculosis. *A**. PCR reactions to confirm the allelic replacement in MtΔmce2R. Primers were designed to amplify either an internal *mce2R* region (Primers WT) or the mutant allele (Primers KO). Molecular weight markers (M) are shown on the left. C- is negative PCR control. The expected molecular weights of the bands are indicated. **B**. Schematic representation of the wild type H37Rv and the mutant MtΔmce2R. The position of each pair of primers is indicated with arrows.

The *mce2R* gene (*Rv0586*) of *M. tuberculosis* H37Rv was knocked out by replacing bases 137 to 617 of the gene with a hygromycin-resistance cassette. The integrity of the resulting *mce2R* mutant strain was then confirmed by polymerase chain reaction (PCR). Figure 1A shows that no amplification product was detected in the mutant strain, with primers that hybridise within the deleted region of *mce2R*, and that a product of approximately 300 bp, corresponding to the central region of *mce2R*, was amplified in the wild-type strain. Using primers that hybridise 980 bp from the 5′ end of *mce2R* and inside the hygromycin resistance genes, an amplicon of expected size (1,150 bp) was detected only in the MtΔmce2R mutant strain. In order to evaluate the effect of the deletion in *mce2R* on the expression of *mce2* operon, changes in mRNA levels were monitored by quantitative real time PCR (RT-qPCR) in the wild type and in the MtΔmce2R mutant strains. Results showed a significant increase in the level of transcription of *yrbE2A* and *mce2A* (Table 1) in the MtΔmce2R mutant strain compared to the wild type during *in vitro* culture (*p* < 0.05), thus confirming that Mce2R acts as a transcriptional repressor of the *mce2* operon. Importantly, the reintroduction of *mce2R* significantly decreased the transcription of the *mce2* genes in the mutant strain (see below). Since our earlier work had shown that *mce2R* and the *mce2* operon are co-transcribed [10], the decreased transcription of the *mce2* genes in the complemented strain further indicates that the upregulation of the *mce2* gene in the knockout mutant was not the result of a polar effect of the disruption of *mce2R* but rather the consequence of a loss of repression by the regulator.

**Table 1:** **Comparison of the gene expression ratios of *mce2 *genes, obtained by RT-qPCR**

**Gene name**	**Fold change MtΔmce2R/H37Rv**		**Fold change MtΔmce2R Comp/H37Rv**	
	**EEP**	**LEP**	**EEP**	**LEP**
MtH37Rv-0587 (*yrbE2A*)	4.95	ND	−2.71*	−5.43
MtH37Rv-0586 (*mce2R*)Ψ	10.14	3.47	29.5	3.99
MtH37Rv-0589 (*mce2A*)	6	ND	ND	ND
MtH37Rv-0590 (*mce2B*)	ND	ND	ND	−4.6

*Values were not statistically different between strains.

ΨPrimers encompass 137 bp of the 5’ end of *Rv0586*, which are conserved in the mutant.

*Abbreviations: ND* not determined, *EEP* early exponential phase, *LEP* late exponential phase.

The values indicate the average ratios of MtΔmce2R/*M. tuberculosis* H37Rv or MtΔmce2R Comp/*M. tuberculosis* H37Rv for four independent biological replicates.

The growth profiles of the wild type, mutant and complemented strains under *in vitro* standard culture conditions showed similar doubling times. Thus, the mutation of the *mce2R* gene does not appear to compromise the *in vitro* growth of *M. tuberculosis* (data not shown).

### Overexpression of Mce2R reduces *M. tuberculosis* replication in a mouse model of infection

**Figure 2:**
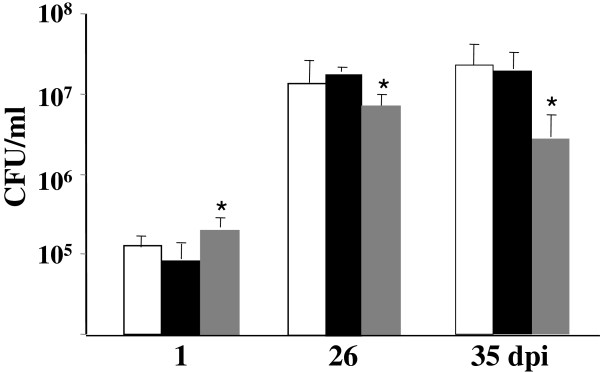
**Replication of the MtΔmce2R mutant, the wild type and complemented strains in mouse lungs after intratracheal inoculation.** Groups of mice were infected by intratracheal injection of wild type (white bars), MtΔmce2R (black bars), MtΔmce2RComp (grey bars). At 1, 26 and 35 days post-infection, mice were sacrificed and viable bacteria present in the lungs were recovered. The results are expressed as the mean number of CFUs ± standard deviations in five mice. These data are based on one of two independent experiments with similar results. *(*p* < 0.05) significantly different from values of the wild type strain.

In order to examine the infection and survival pattern of the MtΔmce2R mutant *in vivo,* we used the intratracheal route to infect BALB/c mice [8], and determined lung colonization by counting bacterial colony forming units (CFUs). At 26 and 35 days post-infection, the number of CFUs in lungs of animals inoculated with the MtΔmce2R mutant was equivalent than that of the animals inoculated with the parental strain (Figure 2). However, the introduction of a constitutively expressed *mce2R* gene into the MtΔmce2R mutant (MtΔmce2RComp) significantly reduced the replication of *M. tuberculosis* in lungs at 26 and 35 days post-infection (*p* < 0.05). This result led us to hypothesize that the expression of the *mce2* operon was over-repressed in the complemented strain due to the overexpression of Mce2R. To test this possibility, we assessed the *in vitro* expression of *mce2R* and *yrbE2A* in the complemented and the wild type strains at both the early and late exponential phases of bacterial growth. The level of transcription of *mce2R* in the complemented strain was higher than in the wild type strain (*p* < 0.05) at the exponential and stationary growth phases (Table 1). At the early exponential phase, the differences in the amount of *yrbE2A* mRNA between both strains were not statistically significant whereas at the late exponential phase there was a significant reduction in *yrbE2A* mRNA (*p* < 0.05) in the complemented strain as compared with that in the wild type strain (Table 1).

### The lack of Mce2R only affects the expression of *mce2* operon during the *in vitro* culture of *M. tuberculosis*

To define the Mce2R regulon we performed a whole-genome *in vitro* expression profiling on the mutant and wild-type parental H37Rv strains. The analysis of gene expression data showed that about 99.6% of all genes showed fold changes equal or greater than 1.2 (absolute value) (Additional file 1: Table S1), indicating that most of the genes were similarly expressed in the mutant and the wild type strains. We found only 16 genes that were overexpressed in MtΔmce2R with fold changes >1.2.

A subset of those genes that were upregulated in the MtΔmce2R mutant strain in the microarray experiments was assessed by RT-qPCR. Only the *mce2* genes were significantly upregulated in the mutant strain (*p* < 0.05, Table 1). The other genes that were overexpressed in the microarray experiment showed even lower and/or non-significant fold changes in the RT-qPCR assays (Additional file 1: Table S1), with the exception of *Rv0324* that was downregulated in both the microarray and RT-qPCR experiments (*p* < 0.02). Altogether, these results indicate that in standard *in vitro* culture conditions Mce2R mainly regulates the expression of the *mce2* operon.

### Overexpression of *mce2R* reduces the arrest of mycobacteria-containing phagosomes

We next evaluated the maturation stage of mycobacterial phagosomes using immunofluorescence and confocal microscopy. *M. tuberculosis* strains were used to infect J774 macrophages for 1 hour of uptake and two hours of chase as described in Material and Methods and processed for microscopy.

**Figure 3:**
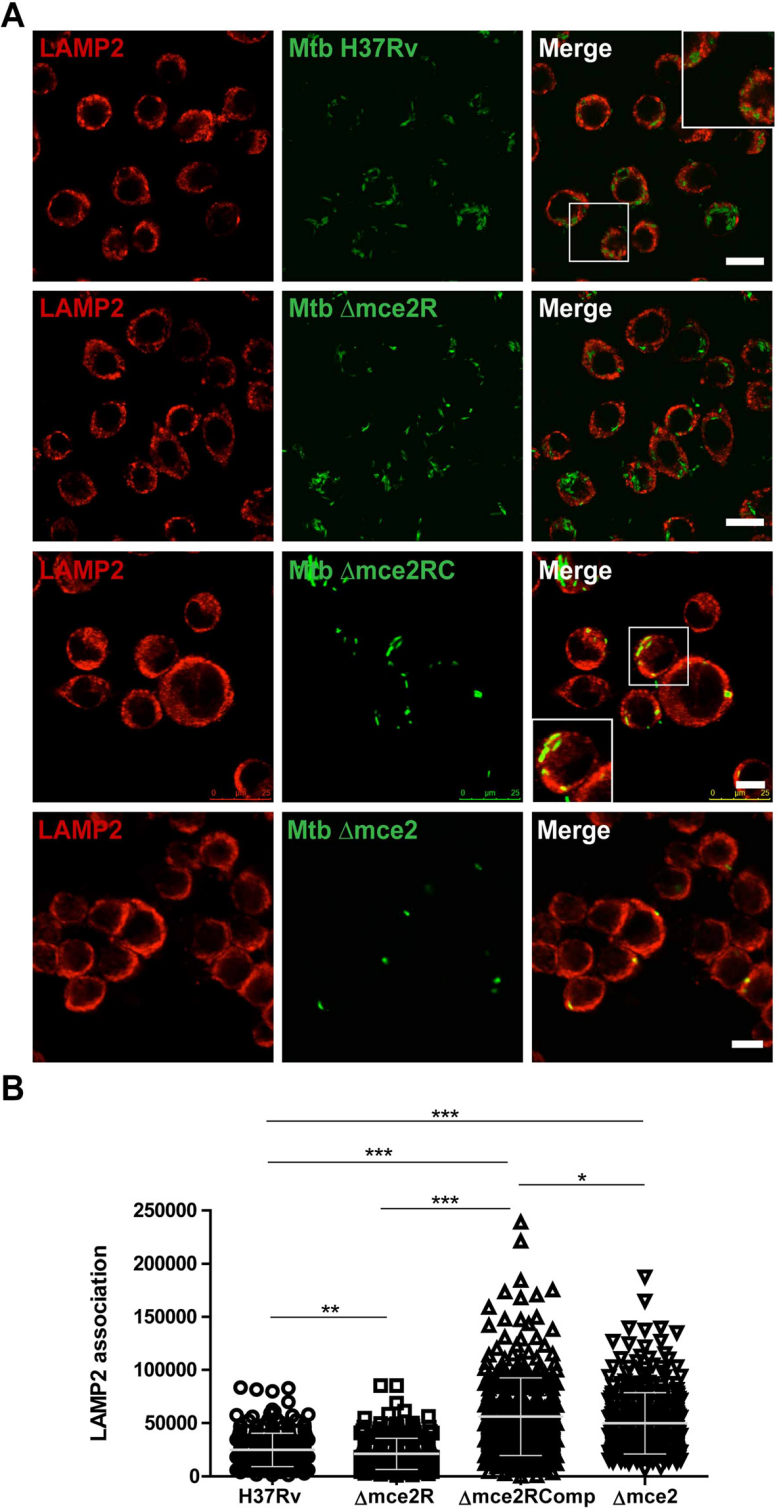
**The overexpression of *mce2R *decreases the arrest of phagosome maduration. A**. LAMP-2 association of *M. tuberculosis* H37Rv, Mt∆mce2R, Mt∆mce2RComp and MtΔmce2-containing phagosome. J774 macrophages were infected with *M. tuberculosis* strains for 1 h, washed and incubated for additional 2 h in RPMI media. Phagosomal LAMP-2 was detected using an appropriate antibody (red) and the bacteria were stained with FITC (green). The cells were analyzed by confocal microscopy and in the Merge box is observed the bacteria-LAMP2 association. Scale bars: 10 μm. **B**. Quantification of that observed in A). These data are based on one of two-four independent experiments with similar results. (***) Indicates significance where *p* < 0.001, (**) where *p* < 0.01 and (*) where *p* < 0.05.

In three of four independent experiments, the fraction of Lysosomal-associated membrane protein 2 (LAMP-2)-positive phagosomes was slightly, but significantly (*p* < 0.01), lower in cells infected with MtΔmce2R, as compared to the wild-type strain (Figure 3). Consistently with the *in vivo* replication experiments, overexpression of Mce2R in the complemented strain significantly increases the maturation of *M. tuberculosis*-containing phagosome (*p* < 0.001). These results suggest that Mce2R regulon participates in the phagosomal arrest induced by intracellular *M. tuberculosis* to survive and replicate inside macrophages [11]. In order to know the contribution of *mce2* operon to the phagosome arresting we evaluated the association of LAMP-2 marker with phagosomes containing a *M. tuberculosis mce2-*knockout (MtΔmce2, [8]). In two independent experiments the number of LAMP-2-positive phagosomes were higher (*p* < 0.001) in cells infected with MtΔmce2 than in those infected with the wild-type strain (Figure 3), indicating that *mce2* operon encodes proteins with a role in phagosome arresting.

## Discussion

In the present study we demonstrated that the knockout of the transcriptional repressor Mce2R does not affect the replication of *M. tuberculosis* in mouse lungs. This finding suggests that similarly to that observed for the *in vitro* growth of *M. tuberculosis*, Mce2R weakly represses the *in vivo* expression of the *mce2* virulence operon, likely due to the fact that this repressor negatively regulates its own expression. Remarkably, when the transcription of *mce2R* was conducted by a strong and desregulated promoter, the resulting complemented strain expressed higher levels of *mce2R* mRNA than the wild type strain, and was significantly more attenuated than the mutant *M. tuberculosis* strain, in terms of bacterial replication in lungs. Thus, these observations may indicate that, during the *in vivo* infection, the expression of the *mce2* operon is more effectively repressed in the complemented strain than in the wild type strain*.* In *in vitro* growth conditions, the expression of *yrbE2A* was significantly repressed in the complemented strain only at the stationary growth phase, suggesting that Mce2R could effectively repress the transcription of the *mce2* operon when a substantial level of this repressor is accumulated. This *in vitro mce2* expression profile supports the hypothesis that increasing bacterial attenuation along the infection is a consequence of an increasing reduction of the expression of the *mce2* operon. Importantly, the results of this study are consistent with previous findings demonstrating that a mutation in the *mce2* operon impairs either the replication or the lethality of *M. tuberculosis* in mouse models [8,9].

We also defined the *in vitro* Mce2R regulon by whole genome microarray analysis and determined that the genes whose expressions were significantly affected by the transcriptional regulator were confined to those belonging to the *mce2* operon. Surprisingly, the expression of the *end* gene, which has been suggested to be regulated by Mce2R [10], showed no changes in expression in the mutant strain compared to the wild type. This difference is probably a reflection of the different experimental setups in each study. While in the present study the conditions used to study gene expression were based on the absence or presence of Mce2R, our previous study investigated the effect of modulating the expression of *mce2R*. The expression *Rv0324*, which encodes a putative transcriptional regulator, was slightly reduced in the mutant strain, suggesting that the lack of Mce2R indirectly affects the expression of *Rv0324*. However, the low fold change detected for this gene in both experimental strategies places in doubt the biological significance of this differential expression. The type of exclusive *in vitro* regulation of Mce2R over the *mce2* operon contrasts to that described for Mce3R, the transcriptional repressor of the *mce3* operon [12,13]. Whereas during the *in vitro* growth of *M. tuberculosis*, Mce3R negatively regulates the expression of two transcriptional units likely to be involved in lipid or isoprenoid modifications [13], Mce2R seems to regulate exclusively the transcription of *mce2*. Our findings thus suggest that the higher virulence of the mutant MtΔmce2R strain (as compared to the *mce2R* overexpressing-complemented strain) is, at least in part, a consequence of the overexpression of the *mce2* operon.

One of the main mechanisms elicited by intracellular mycobacteria to survive and replicate inside the host cells is to arrest the normal process of phagosome maturation, which enables bacterial survival in a non-acidified intracellular compartment [11]. Proteins involved in the biosynthesis of cell wall lipids, such as PhoP [14] and Ag85A [15], have shown to have a role in the phagosome arresting exerted by *M. tuberculosis*. Likely, these proteins are not direct modulators of phagosome trafficking, instead they would participate in the synthesis of compounds that are actually implicated in this cellular process. For instance, the synthesis of cell wall trehalose dimycolate and the sulfolipids is regulated by the two-component system PhoP/PhoR and these lipids have been described as implicated in blocking phagosome/lysosome fusion induced by *M. tuberculosis*[11]. However, a recent report has suggested the opposite, showing that overproduction of the sulfoglycolipids (SGL), Ac3SGL and Ac4SGL in the *M. tuberculosis* Rv1503c::Tn and Rv1506c::Tn strains increases the intracellular trafficking to lysosomes of these mutant strains. In connection with this last finding, previous reports have suggested a role of the proteins encoded in the *mce2* operon in the sulpholipid metabolism/transport. Firstly, Marjanovic et al. have shown that a *M. tuberculosis* deleted in *mce2* operon accumulates more sulpholipids (SLs) than it parental H37Rv strain, proposing that the *mce2* operon encodes proteins involved in the metabolism/transport of SLs [16]. Secondly, the finding that sigma factor L seems to regulate the expression of *mce2* genes and genes encoding enzymes implicated in SL synthesis and the fact that the *mce2* operon is absent in *Mycobacterium smegmatis*[4], which does not produce SL-1 [17], also support a role of Mce2 proteins in the transport of SLs. Based on these previous observations and the results of this study, we can speculate that lack of Mce2 proteins (either by mutation or over-repression) increases the accumulation of SLs in the bacteria, disfavouring the arrest of phagosome maturation and in turn the survival of both the mutant MtΔmce2 [8] and the complemented MtΔmce2Comp in mouse lungs. However, the higher maturation of phagosomes containing the over-repressed strain (MtΔmce2RComp) as compared to that of phagosomes containing MtΔmce2 (*p* < 0.05) may indicate that other *in vivo* Mce2R-regulated genes can also participate in the phagosome arresting induced by intracellular *M. tuberculosis*. Whether the mutation of *mce2R* affects the accumulation of SLs in *M. tuberculosis* will require further investigation and is beyond the scope of the present study.

## Conclusions

The results of the present study demonstrate that, in standard conditions of *in vitro* culture, Mce2R mainly regulates the expression of the *mce2* operon and support a role of the proteins regulated by this transcriptional repressor in the virulence of *M. tuberculosis*. In addition, we showed that this role in the virulence for Mce2R regulon takes place in part through interfering with the normal maturation of phagosomes. However, further research is needed to better understand the mechanisms of regulation exerted by Mce2R and the role of Mce2R regulon in the survival of *M. tuberculosis* inside the host.

## Methods

### Ethical statement

Animal experimentations were performed inside the biosafety facilities of the National Institute of Agricultural Technology (INTA), Argentina, in compliance with the regulations of Institutional Animal Care and Use Committee (CICUAE) of INTA (file number: 31-20-12). CICUAE’s members: Florestán Maliandi (President), Alejandra Romera (Secretary), Marisa Farber, Analía Berinstein, Pablo Chacana, Gabriel Pinto, Bibiana Brihuega, Gisella Marcoppido, Verónica Maldonado May, Lucas Vagnoni, Osvaldo Zabal and Luis Samartino (Vocals).

### Bacterial strains and culture media

All cloning steps were performed in *Escherichia coli* HB101. *E. coli* were grown either in Luria-Bertani (LB) broth or on LB agar. *M. tuberculosis* strains were grown in Middlebrook 7H9 medium supplemented with albumin 0.5%, dextrose 0.4%, and glycerol 0.5% (M7H9-AD-G) and either Tween 80 0.05% or Middlebrook 7H11, supplemented with albumin, dextrose and glycerol. When necessary, either 50 μg/ml hygromycin or 20 μg/ml kanamycin was added to the media.

### Construction of *M. tuberculosis* Δmce2R mutant and complemented strains

A mutant strain of *M. tuberculosis* carrying a chromosomal deletion encompassing the bases 137–617 of the *mce2R* (*Rv0586*) gene was obtained by using the gene knockout system described by Bardarov [18]. Briefly, two DNA fragments of approximately 1 kb flanking the 5′ and 3′ regions of *mce2R* were obtained by PCR using *M. tuberculosis* H37Rv genomic DNA as template and the following sets of primers: Regionup-up (tctagaccgtacaactcgatcaat)/Regionup-low (tctagaactccgagcaactcagcc) and Regionlow-up (actagtatctgctcaggtgatccc)/Regionlow-low (actagtacgccgatcgtggtcaac). Flanking arms were directionally cloned into *Xba*I and *Spe*I sites of cosmid pYUB854 [14]. The recombinant cosmid was digested by *Pac*I and ligated to *Pac*I-digested concatemerized DNA of phage phAE87. To generate each specialized transducing phage, the *Pac*I-digested recombinant cosmid was used to replace cosmid pYUB328 in phAE87 an *in vitro* λ-packaging reaction (GIGAPackII, Stratagene). After transducing *E. coli* HB101 and plating the transductants on selective media containing hygromycin. Phasmid DNA was prepared from the pooled antibiotic-resistant transductants and electroporated into *M. smegmatis* mc^2^155. Transductants were grown at the permissive temperature of 31°C to allow phage replication, and then transducing phages were prepared from isolated plaques as previously described [18]. Transducing phages, carrying the mutated allele of *mce2R* were used to infect *M. tuberculosis* H37Rv as previously described [18]. Infected mycobacteria were plated onto media containing hygromycin at the restrictive temperature of 37°C. Colonies that appeared after 25 days of culturing were analysed by PCR for the presence of the deletion in the *mce2R* gene. Only one clone showed a 480-bp deletion from *mce2R* and was referred to as MtΔmce2R. Deletion of *mce2R* in MtΔmce2R strain was confirmed by PCR analysis using two sets of primers: one set that hybridises inside *mce2R* (WT-forward: gatctgttgccccgattgt/WT-reverse: tctacgatcgaaccggcgct), and the other that hybridises at approximately 1000 bp from the 5′ ends of both *mce2R* and inside the hygromycin resistance gene (KO-forward [Low new2R] acgtcagcttcagccagagt, KO-reverse [5′hygro-reverse]: tcagcaacaccttcttcacg).

In order to complemente the mutant phenotype, a fragment containing *mce2R* gene was amplified by PCR using the primers up mce2r pw16 (catatgatctgttgccccgattgttgt) and low mce2r pw16 (catatgcattgccgactcgcct), and cloned into pW16 to produce plasmid pW16mce2R. This plasmid was used to transform the *M. tuberculosis* MtΔmce2R strain by electroporation to produce the complemented strain MtΔmce2RComp.

### Mouse infections

The experimental BALB/c model of progressive pulmonary tuberculosis has been previously described in detail [8]. Briefly, bacillary suspensions were adjusted to 1.25 × 10^5^ viable cells in 100 μl phosphate buffer saline (PBS). Each animal was anesthetized and intratracheally inoculated with *M. tuberculosis* strains. Infected mice were kept in cages fitted with microisolators connected to negative pressure. Groups of 15 mice were each infected with the different *M. tuberculosis* strains. The inoculum doses were determined by enumerating the CFUs recovered from the lungs of five mice per infection strain 24 h post-infection. Five mice per group were killed at 1, 26 and 35 days after infection and lungs removed and homogenized. Four dilutions of each homogenate were spread onto duplicate plates. This experiment was repeated twice with similar results. Animal experimentations were performed inside the biosafety facilities of the National Institute of Agricultural Technology (INTA), Argentina, in compliance with the regulations of Institutional Animal Care and Use Committee (CICUAE) of INTA. Student’s *t* test was used to determine significant differences between groups.

### Macrophage infections

*M. tuberculosis* strains were cultivated until exponential growth phase, pelleted, washed twice in PBS and re-suspended in RPMI medium to a multiplicity of infection (m.o.i.) of 5. Clumps were removed by ultrasonic treatment in a water bath followed by a low speed centrifugation for 2 min. Macrophages were seeded into 24 well tissue culture plates at 80% confluence and infected for 1 hour (uptake). Afterwards, cells were washed and incubated in full medium for another 2 hours (chase).

### Inmunofluorescense and confocal microscopy

For indirect immunofluorescence, *M. tuberculosis* strains treated as described above, were covalently stained with FITC (isomer I; Sigma. FITC solution was prepared 20 mg/ml in DMSO). Briefly, 1 × 10^9^ bacteria were washed twice with 0.1 M buffer Na_2_CO_3_/NaHCO_3_ (pH 9) and suspended in 1 ml of the same solution. FITC was added to a final concentration of 1 mg/ml and incubated in the dark for 2 h at 37°C. Bacteria were washed gently with PBS until unbound colorant was eliminated, and used to infect J774 macrophages as was described above. Infected cells were fixed with 3% paraformaldehyde solution in PBS for 20 min and quenched by incubating with 50 mM glycine solution for 10 min. Then, cells were permeabilized with 0.05% saponin in PBS containing 0.2% BSA for 15 min, and incubated with the primary anti-LAMP-2 (ABL-93, DSHB) antibodies diluted 1:50 in PBS. anti-LAMP-2 antibodies were obtained from the Developmental Studies Hybridoma Bank, developed under the auspices of the NICHD and maintained by The University of Iowa, Department of Biology, Iowa City, IA 52242. Secondary antibodies anti-Rat Cy5-conjugated (Jackson Immuno Research Labs Inc.) was used diluted 1:600 in PBS. Each step with antibodies was incubated for 1 hour. Cells were mounted with Dako mounting media (Dako, Denmark) and analysed by confocal microscopy using a Leica SP5 AOBS confocal microscope (Leica Microsystems, Germany). Internalization of the mycobacteria was followed through the fluorescence of green FITC and the LAMP-2 association to mycobacterial phagosomes was counted in at least 50 cells using Fiji/ImageJ program (U.S. National Institute of Health, Bethesda, Maryland, USA). The analysis was performed for duplicates in three-four independent experiments. Statistical determinations were made using *t* test.

### RNA preparation

DNA-free RNA was extracted from 50 ml mid-exponential-phase cultures of *M. tuberculosis* as described by Santangelo et al. (2002) [12].

Prehybridisation, hybridisation, and washing steps were performed as described previously [13,19]. Microarrays were hybridised with a combination of Cy3-cDNA generated from genomic DNA of *M. tuberculosis* H37Rv and Cy5-cDNA obtained from total RNA of either *M. tuberculosis* H37Rv or MtΔmce2R.

Eight sets of microarray data, consisting of eight biological replicates (cells from independent cultures), were produced for each *M. tuberculosis* strain.

The microarrays were scanned using an Affymetrix 428 scanner and fluorescent spot intensities were quantified using BlueFuse for Microarrays v3.2 (BlueGnome Limited, http://www.cambridgebluegnome.com). For each spot, background fluorescence was subtracted from the average spot fluorescence to produce a channel specific ratio.

### Data processing and statistical analysis

Log2 Cy5:Cy3 (test:control) ratios were used for subsequent calculations. Within each microarray, block median normalisation, excluding control and empty spots, was carried out using the BlueFuse software. Median absolute deviation using Mathematica 5.2 (Wolfram Research Inc.) was applied to bring the histograms of all microarrays into the same scale. Technical replicates were averaged. Differentially expressed genes between the strains were detected by applying t-tests with a Benjamini and Hochberg adjusted p-value correction.

### RT-qPCR

RT-qPCR reactions were performed as described by Santangelo et al. [13,20] using DNA-free RNA (1 μg) extracted from mid-exponential growth-phase cultures and specific primers. Relative quantification was performed by using *sigA* as a reference gene and a subsequent analysis for statistical significance of the derived results was performed by using the Pair Wise Fixed Reallocation Randomization test [21]. The mean value of PCR efficiency for the primers (Additional file 2: Table S2) was 92% to 100%. These values were calculated using both the classical dilution curve and slope calculation (E = 10 [−1/slope] − 1) [21] and an estimation by absolute fluorescence increase [22].

## Competing interests

The authors declare that they have no competing interests.

## Authors’ contributions

MAF carried out the confocal studies and immunoassays, and drafted the manuscript. MVB carried out the infections, FCB carried out the RT-qPCR, JN and MS carried out the statistical analysis, MPS constructed the mutant strain, RVR constructed the complemented strain, CLV and MGG participated in the design of the study, PG and LIK performed the microarray study analysis, FB conceived of the study, and participated in its design and coordination and helped to draft the manuscript. All authors read and approved the final manuscript.

## Supplementary Material

Additional file 1: Table S1 Differential expressed genes between MtΔmce2R/*M. tuberculosis* H37Rv. Click here for file

Additional file 2: Table S2 Primers used in RT-qPCR. Click here for file
