# Supplementary material for: Study of the in vivo role of Mce2R, the transcriptional regulator of mce2 operon in Mycobacterium tuberculosis
Source: BMC Microbiol. 2013 Sep 5;13:200. doi: 10.1186/1471-2180-13-200 (PMC3847441; doi:10.1186/1471-2180-13-200)
Supplement: Additional file 1: Table S1 — Differential expressed genes between MtΔmce2R/M. tuberculosis H37Rv. [file 1471-2180-13-200-S1.docx]

**Differential expressed genes between MtΔmce2R / *M. tuberculosis* H37Rv**

| Rv number | Fold change  Microarray | Adj. P Val | Fold change  RT-qPCR* |
| --- | --- | --- | --- |
| Rv0586** | 1.412 | 0.0223 | 10.14* |
| Rv3467 | -1.226 | 0.0223 | -1.283 |
| Rv2649 | -1.525 | 0.0223 |  |
| Rv0324 | -1.552 | 0.0223 | -1.436* |
| Rv3781 | -1.628 | 0.0223 | -1.021 |
| Rv3761c | -1.475 | 0.0307 | -1.032 |
| Rv1375 | 1.443 | 0.0380 |  |
| Rv3472 | -1.250 | 0.0380 |  |
| Rv3042c | 1.420 | 0.0393 |  |
| Rv0812 | 1.369 | 0.0393 |  |
| Rv1563c | -1.210 | 0.0439 |  |
| Rv0591 | 1.431 | 0.0466 |  |
| Rv2619c | -1.616 | 0.0475 |  |
| Rv2331A | 1.514 | 0.0485 |  |
| Rv0697 | 1.329 | 0.0485 | -1.231 |
| Rv3915 | -1.882 | 0.0485 | -1.118 |

* Statistically different

**The probe in the microarray encompasses only the first 100 bp of *Rv0586,* which are conserved in the mutant.
