# Supplementary material for: Study of the in vivo role of Mce2R, the transcriptional regulator of mce2 operon in Mycobacterium tuberculosis
Source: BMC Microbiol. 2013 Sep 5;13:200. doi: 10.1186/1471-2180-13-200 (PMC3847441; doi:10.1186/1471-2180-13-200)
Supplement: Additional file 2: Table S2 — Primers used in RT-qPCR. [file 1471-2180-13-200-S2.docx]

**Primers used in RT-qPCR.**

| **Gene name** | **Sequences** |
| --- | --- |
| *yrbE2A* (*Rv0587*) | F: TCTACCGGACATGCGTACTG  R: CAGCAGGATATTGAGCGTGA |
| *mce2R* (*Rv0586*) | F: ATGGCGCTGCAGCCGGTGACT  R: ACTCCGAGCAACTCAGC |
| *mce2A* (*Rv0589*) | F: GAAGACCGAGCTGACTATGG  R: ATGTAGCGAGGATTCACGTC |
| *mce2B* (*Rv0590*) | F: CCCACTCTTCCAAACGTTGG  R: CGAGGATGTCGTTGATGGTG |
